# Supplementary material for: Treatment outcomes and prognostic factors in external auditory canal squamous cell carcinoma
Source: PLoS One. 2025 Dec 29;20(12):e0339860. doi: 10.1371/journal.pone.0339860 (PMC12747381; doi:10.1371/journal.pone.0339860)
Supplement: Attachment — Submitted filename: Responses to reviewers.docx [file pone.0339860.s003.docx]

**Responses to Reviewers**

On behalf of the authors, I would like to thank you and the reviewers for the valuable and helpful comments on our submitted manuscript, “Treatment outcomes and prognostic factors in external auditory canal squamous cell carcinoma."
We have carefully reviewed the comments and revised the manuscript as appropriate. Our responses are provided point by point, and changes to the manuscript are highlighted in yellow.

**Reviewer #1**: In this manuscript entitled “Treatment Outcomes and Prognostic Factors in External Auditory Canal Squamous Cell Carcinoma”, written by Kang et al, the authors retrospectively reviewed clinical, pathological and survival data of 56 patients with External Auditory Canal Squamous Cell Carcinoma (EACC). The authors performed survival analysis as well as multivariate analysis. The statistical methods in this study are mostly appropriate and the authors discussed their results comparing the previous studies of EACC. Overall, the manuscript is in good quality. I have few comments below.

1) Figure 1A and 1B. Please include the table for "number at risk" under the Kaplan-Meier curves so that the readers easily understand the changes in the patient numbers tested. Also, censoring points should be indicated in the plots.
I strongly recommend the authors include T1 and T2 patients in the Kaplan Meier plots as well. Since the authors noted the survival rate for T1 and T2 patients was 100%, including these groups in the figure may indicate a rationale for the good quality of treatment the authors provided.

**Response:**

We thank the reviewer for this valuable suggestion. In response, we have revised our survival figures to improve clarity. Since overall survival (OS) and disease-specific survival (DSS) curves differed in only two patients, **we integrated them for concise presentation and now provide OS as the representative survival metric**. This unified OS curve offers a more streamlined and interpretable visualization without loss of clinical information.

Accordingly, we have:

- Combined the OS and DSS plots into a **single Figure 1**
- Included **T1** and **T2** patients in the analysis, demonstrating the 100% survival in early-stage disease
- Added **number-at-risk tables** beneath the Kaplan–Meier curves
- Marked **censoring points** for interpretability

These changes strengthen the clarity of the data while fully reflecting the reviewer’s recommendations.

2) Figure 2. How did the authors divide the patients by age into “young” and ‘’old” for univariate and multivariate analyses? I could not find the cutoff for age in these analyses.

**Response:**

We appreciate the reviewer’s comment. In our analysis, **age was treated as a continuous variable**, and we did not dichotomize the patients into “young” and “old” groups. Therefore, the hazard ratio in the univariate and multivariate analyses represents the **incremental increase in risk associated with increasing age**, rather than a comparison between predefined age categories. We have clarified this point in the revised Methods & Results section.

3) Recent clinical and translational research (Tsunoda et al, Laryngoscope Investig Otolaryngol. 2017, PMID: 28894818; Sato et al, Cancer Sci. 2020, PMID: 32500594) indicated that habitual ear picking and subsequent chronic inflammation could be a common risk for EACC (I assume that this would be particularly in eastern Asian population). Did the authors investigate potential risk factors and/or clinical backgrounds in these patients? The authors may discuss this point citing the publications above.

**Response:**

We appreciate this insightful suggestion. Unfortunately, information regarding habitual ear picking was not systematically recorded in the patients’ medical records and thus could not be analyzed in this retrospective study. We have, however, added a paragraph in the **Discussion** citing *Tsunoda et al.* (2017) and *Sato et al.* (2020), which proposed an association between habitual ear picking, chronic inflammation, and the development of EACC. It should be noted that the causal relationship remains uncertain—habitual ear picking may act as a contributing factor, but it may also arise as a consequence of underlying chronic irritation or early neoplastic changes.

**Reviewer #2:** This paper is a retrospective review of 56 cases of squamous cell carcinoma of the external auditory canal. Focusing on squamous cell carcinoma alone is a strength of the paper. That early-stage tumors (T1 and T2) outnumbered advanced stage disease is an unusual feature of the study population. IRB approval was obtained for this study. Inclusion and exclusion criteria are outlined. The modified Pittsburgh classification was used for staging. The paper is well written.
There are significant deficiencies in this paper. The authors do not detail the adjuvant therapy given to this cohort. Under Methods, the authors mention that “**Adjuvant radiotherapy was administered in cases with positive margins, perineural invasion, or advanced disease features,”** but we are not told who had positive margins, etc. Under Surgical Methods and Survival Outcomes, the authors write that none of the T1 or T2 patients received radiotherapy, while all T3 and T4 patients received postoperative chemoradiotherapy. The chemotherapy agents are not mentioned. The radiotherapy doses are not mentioned.
The authors should provide more details regarding the entire cohort of patients. In Table 2, the authors provide details on T4 patients; this level of detail should be provided for all patients.

**Response:**

We appreciate the reviewer’s valuable suggestion. We have expanded both the **Methods** and **Results** sections to provide further details on adjuvant therapy and patient characteristics across all tumor stages.
The statement in the Methods, “*Adjuvant radiotherapy was administered in cases with positive margins, perineural invasion, or advanced disease features*,” represents the general treatment principle at our institution rather than the exact management provided to each patient.
In our cohort, T1 and T2 tumors were fully resected with adequate surgical margins, and selective neck dissection (levels I–II) revealed no nodal involvement; therefore, no adjuvant therapy was administered to these patients.
Among T3 and T4 cases, radiotherapy (RT) was performed in 24 patients, with total doses ranging from 5,040 to 6,660 cGy over 7–8 weeks. Chemotherapy was administered to 21 patients; however, due to the 20-year span of the cohort and the fact that many patients received systemic therapy at local affiliated hospitals, the specific chemotherapy protocols were heterogeneous and not standardized (mostly cisplatin-based regimens).
Given this variability, direct comparison of chemotherapy regimens was deemed clinically inappropriate and unlikely to yield meaningful statistical interpretation for prognostic analysis.
Two patients did not undergo RT and two patients did not receive chemotherapy due to poor general condition, advanced age, or treatment refusal.
These clarifications have been incorporated into the revised **Methods**, **Results**, and **Discussion** sections.
We have retained **Table 2** to present the detailed characteristics of T4 patients, who represent the most advanced and clinically heterogeneous subgroup. Because the extent and pattern of adjacent structure invasion differ substantially between T stages, comparison of specific anatomical invasion sites (e.g., TMJ, mastoid, dura, carotid abutment) is most clinically meaningful within T4 disease only.
To improve transparency and provide a broader cohort overview, a summary of clinicopathologic and treatment characteristics for all 56 patients (T1–T4) has been added to the Results section.

Suggestions, questions, and comments:
1. The Introduction is too long. It should be no longer than 3 paragraphs. The Introduction should contain the salient facts leading to the study, especially noting the controversies or innovations.

**Response:**

We thank the reviewer for this valuable suggestion. The Introduction has been substantially condensed and reorganized into **three concise paragraphs** to improve clarity and focus. The revised version now:

1. Summarizes the clinical background and standard treatment principles of EACC;
2. Highlights the limitations of previous studies, including inconsistent prognostic factors and unsatisfactory survival despite aggressive multimodal therapy; and
3. States the specific **aim and novelty** of the present study, emphasizing the evaluation of prognostic factors and anatomical invasion patterns in a relatively large, homogeneous cohort of EAC SCC.

These revisions ensure that the **Introduction** presents only the most relevant background, the current controversies, and the rationale for the study in a concise manner.

2. Surgical Methods and Survival Outcomes, it is unclear why the authors tabulated only the T4 cases. Such granular data for all study patients would be of interest to researchers worldwide. For this reviewer, comparing the outcomes of patients who underwent sleeve resection to those who underwent lateral temporal bone resection would be of significant value.

**Response:**

As suggested, the **Results** section now includes a comparison between **lateral temporal bone resection (LTBR)** and **sleeve resection**. Both procedures achieved 100 % 5-year survival, showing no apparent difference in overall outcomes. However, except for one case, all sleeve resections were performed for limited superficial lesions at the EAC entrance. One T2 lesion was treated with sleeve resection at the patient’s request with a favorable outcome, but **LTBR remains the recommended procedure for T2 or more extensive disease**.
In addition, we clarified in the **Results** that detailed comparisons of anatomical invasion were limited to **T4 lesions** because invasion patterns differ substantially by T stage and are most clinically informative in advanced tumors. A summary of clinicopathologic and treatment characteristics for the entire cohort (T1–T4) was also added for context.

3. Table 2 does not mention any adjuvant treatment for this T4 tumors. Did any of these patients receive either chemotherapy or radiotherapy?

**Response:**

We thank the reviewer for this helpful comment. We agree that detailed information on adjuvant therapy is important for interpreting treatment outcomes. Rather than adding these data to Table 2, we have incorporated them into the **Results** and **Discussion** sections for clarity and completeness. Adjuvant therapy was generally recommended according to institutional principles; however, some patients did not undergo radiotherapy or chemotherapy because of poor general condition, advanced age, or treatment refusal. Moreover, due to the 20-year study period and the involvement of multiple affiliated hospitals, chemotherapy regimens varied considerably (though most were cisplatin-based), and radiotherapy doses ranged from 5,040 to 6,660 cGy. Given this variability, we considered a descriptive summary in the text to be more informative than including heterogeneous data in Table 2. These details and explanations have been added accordingly to the **Results** and **Discussion** sections.

**Reviewer #3:** The article is potentially interesting, since it provides a detailed description of the survival outcomes of a homogeneous series of SCC of the external acoustic canal.
The most relevant strength of this article resides in a clear description of the characteristics of the included subjects, and of the anatomical sites of invasion in the advanced-stage cases.
However, it should be more clearly explained what the results of this article add to the current state of knowledge on this field.
Although Authors provided a detailed calculation of the mortality risk (both in univariate and multivariate setting) for each demographic and clinical variable (including a detailed breakdown of the prognostic role of different anatomical invasion sites), the presented data seem to confirm what was already known from the literature (in example, doi: 10.1002/hed.2643; doi: 10.1177/019459989110500102; doi: 10.1055/s-0030-1253575 etc.).
For this reason, I suggest to expand the discussion, developing more in-depth the actual meaning of this results with reference to the existing literature (including the existing different staging methods and comprehensive prognostic scores).

**Response:**

We thank the reviewer for these insightful comments. We have expanded the Discussion to emphasize both the methodological and clinical novelty of our work.
First, our cohort represents one of the largest single-institution series of *external auditory canal squamous cell carcinoma* (EAC SCC) spanning over two decades, including a substantial number of advanced-stage (T3–T4) cases in a disease that is exceedingly rare. This allowed a statistically meaningful analysis of prognostic factors within the advanced subgroup, which has been underrepresented in prior literature. While our findings are consistent with established staging-based prognostic trends, the inclusion of a large number of advanced-stage cases and detailed anatomic correlation analyses provide refined insights that extend beyond prior descriptive reports.
Second, while previous studies—such as those by Arriaga et al. (1991) and Gaudet et al. (2010)—validated the Modified Pittsburgh staging system and its radiologic–pathologic correlation, our analysis extends these frameworks by quantifying survival differences according to specific anatomical invasion sites (e.g., TMJ, mastoid, dura). This detailed survival stratification within advanced disease provides finer prognostic discrimination and may help refine future staging or prognostic models.
Together, these elements highlight the distinct contribution of our study in advancing the understanding of prognostic heterogeneity in this rare malignancy.

Minor concerns:
- does "CA" mean carotid artery involvement in Tab. 2? Please, explain it in the abbreviations.

**Response:**

Yes, we have clarified in Table 2 abbreviations that “CA” refers to carotid artery involvement.

- maybe including the KM graphs also for T1 and T2 cases may improve Fig. 1A and B

**Response:**

: As suggested, we have included Kaplan-Meier graphs for T1 and T2 patients in Figure 1.
