# Supplementary material for: Treatment outcomes and prognostic factors in external auditory canal squamous cell carcinoma
Source: PLoS One. 2025 Dec 29;20(12):e0339860. doi: 10.1371/journal.pone.0339860 (PMC12747381; doi:10.1371/journal.pone.0339860)
Supplement: Attachment — Submitted filename: Responses to Reviewers_2nd.docx [file pone.0339860.s004.docx]

**Responses to Reviewers**

On behalf of the authors, I would like to thank you and the reviewers for the valuable and helpful comments on our submitted manuscript, “Treatment outcomes and prognostic factors in external auditory canal squamous cell carcinoma."
We have carefully reviewed the comments and revised the manuscript as appropriate. Our responses are provided point by point, and changes to the manuscript are highlighted in yellow.

Reviewer #1: Thank you for responding to my questions. All of my questions have been solved properly, and the quality of the manuscript has been significantly improved.

Reviewer #4: Jong Woo Chung et al. present a retrospective study of 56 cases of external auditory canal squamous cell carcinoma (EAC-SCC) treated with surgical intervention. The relatively large number of cases and the follow-up data after treatment make this study a potentially valuable contribution. As a consolidated case series from China, the report has significance; however, it unfortunately lacks novelty. Moreover, essential information typically expected in a case series is not sufficiently provided.

Minor Points:

#1. The number of significant digits used in the manuscript is inconsistent and should be standardized.

**Response:**

We carefully reviewed all numerical values in the manuscript and standardized significant digits across the text and tables. Hazard ratios are reported to two decimal places, percentages to one decimal place, and p-values to three decimal places. In Figure 2, p-values are displayed with two decimal places for visual clarity, which consists of common graphical presentation practices.

#2. It should be clearly stated whether the TNM staging presented is clinical or pathological.

**Response:**

We agree that this requires clarification. Surgical planning was based on clinical staging using the modified Pittsburgh classification system, whereas final tumor staging for analysis was determined by postoperative pathological assessment. We clarified this in the Methods section. We also described in the Results that two cases initially staged clinically as T2 were downstaged to pathological T1 based on histopathological confirmation of the absence of true bone erosion.

Major Points:

#1. Whether the surgeries were performed via en bloc resection or piecemeal resection is critically important for oncologic discussion and must be described explicitly.

**Response:**

We thank the reviewer for this important comment. We have clarified the surgical technique in the Methods and Results sections.

All surgical resections were planned and performed with the intent of en bloc tumor removal. Complete en bloc resection was feasible for all T1–T3 tumors. In T4 cases, initial en bloc resection was attempted; however, when invasion involved critical adjacent structures (including the dura, carotid abutment, jugular bulb abutment, or Eustachian tube involvement), additional stepwise resection was performed following en bloc removal to achieve maximal tumor clearance.

This clarification has been added to the revised manuscript.

#2. The manuscript states: “For T3 and T4 lesions, all patients underwent postoperative adjuvant chemoradiotherapy (CRT).” However, administering postoperative radiation therapy in all advanced cases, even when surgical margins are negative, may constitute overtreatment from an oncologic perspective.

**Response:**

We respectfully disagree with the concern regarding potential overtreatment.

In patients with locally advanced EAC SCC (T3–T4), postoperative radiotherapy is widely regarded as standard practice even after margin-negative resection, primarily to improve local control due to the infiltrative growth patterns and high risk of microscopic residual disease. Previous studies, including those by Arriaga et al. and Gaudet et al., have consistently demonstrated that advanced-stage tumors are treated with adjuvant RT irrespective of margin status, and this strategy is considered part of accepted multimodal management rather than overtreatment.

Accordingly, in our cohort, adjuvant therapy was administered using a risk-adapted approach following established treatment principles for advanced disease rather than applied indiscriminately.

We have clarified our institutional principles for postoperative adjuvant therapy in the Methods section using a risk-adapted framework. We have also added a dedicated subsection in the Results detailing the actual treatment patterns, radiation doses, chemotherapy regimens, and the clinical circumstances in which adjuvant therapy was not administered.

#3. Data on postoperative margin status are lacking. Margin assessment is essential information. It is problematic to engage in discussion regarding oncologic outcomes without presenting data on surgical margins. At a minimum, margin status should be included in the case profile of T4 patients (Table 2).

**Response:**

We thank the reviewer for emphasizing the importance of margin assessment.

We have now included surgical margin data for T4 patients in Table 2. Among the 19 T4 cases, negative margins on the initial en bloc main specimen were achieved in 5 patients, while 14 patients showed positive margins on the primary specimen, mostly due to tumor involvement adjacent to critical structures such as the carotid artery, jugular bulb, or tympanic cavity.

Importantly, positive margins referred to the initial resection specimen only. In these cases, additional stepwise excision was performed after en bloc removal, guided by intraoperative frozen-section analysis until tumor clearance was achieved at all accessible margins. For areas adjacent to the carotid artery or jugular bulb, macroscopic assessment was used. No patients demonstrated true intraluminal invasion of these vascular structures.

These clarifications and corresponding data have been added to the Results section, Discussion section, and Table 2 in the revised manuscript.

#4. The rationale for performing surgery on cases with preoperative imaging suggesting carotid artery (CA) involvement should be clarified. According to existing literature, complete resection with negative margins is theoretically impossible in such cases, and curative treatment is considered extremely difficult even with adjuvant therapy. Given that all such patients in this series ultimately died, the justification for surgical intervention in these cases should be discussed in detail.

**Response:**

We thank the reviewer for this important comment regarding surgery in cases with suspected carotid artery (CA) involvement.

We have clarified in the Discussion that preoperative imaging findings in our cohort represented CA abutment rather than true intramural or intraluminal invasion. No cases required carotid sacrifice or demonstrated destructive vascular wall invasion. In such cases, en bloc tumor resection was followed by additional stepwise excision and careful scraping of adherent tumor tissue from the CA surface until macroscopic clearance was achieved, with curative intent.

Therefore, these procedures were not palliative but undertaken to achieve maximal oncologic clearance. The universally poor outcomes observed in these patients reflect the aggressive biological behavior of advanced-stage disease with extensive skull base or intracranial involvement rather than the futility of surgical management itself. Corresponding clarification has been added to the revised Discussion.

#5. The manuscript distinguishes between jugular fossa and jugular bulb invasion in preoperative imaging. However, is it truly feasible to differentiate these two sites accurately based on imaging alone? The basis for this distinction should be clarified. If jugular bulb invasion was diagnosed preoperatively, resection of the involved vein should have been planned in order to achieve negative margins. In practice, direct visual confirmation of venous invasion is not possible intraoperatively, as the tumor would already be exposed at that point. Despite this, the Discussion section states: “no cases involved direct invasion of the carotid artery or jugular bulb,” which appears contradictory if the preoperative strategy was to achieve negative margins.

**Response:**

We thank the reviewer for this important clarification regarding jugular fossa and jugular bulb involvement.

We have revised the Discussion to clearly define our terminology and diagnostic approach. Jugular fossa involvement was defined as bony skull-base invasion, which is reliably identified by preoperative imaging. Jugular bulb “involvement” referred to venous surface abutment or adherence without destruction of the venous wall or intraluminal extension. No cases demonstrated true jugular bulb invasion requiring venous sacrifice or resection.

In cases of jugular bulb abutment, careful surface excision and scraping were performed intraoperatively to achieve macroscopic clearance; one patient required limited surface reconstruction using absorbable hemostatic material. The statement “no cases involved direct invasion of the jugular bulb” refers specifically to the absence of intraluminal venous invasion and is therefore not contradictory to our surgical management strategy. Corresponding clarification has been added to the revised Discussion.

Reviewer #5: This paper investigates prognostic factors for external auditory canal carcinoma, focusing mainly on the impact of invasion sites in T4 cases. Although similar studies have been published previously and no new prognostic factors were identified, the paper holds value as a case series.

There are several points of concern that require revision, which I have listed below.

1) In surgical procedure section, line 8. Does this mean that neck dissection was performed in all T1 and T2 cases, but no lymph node metastasis was found in any of them? At the same time, it is also necessary to describe how the parotid gland was managed. Additionally, please indicate whether neck dissection and parotidectomy were performed in T3 and T4 cases.

**Response:**

We appreciate the reviewer’s thoughtful comment regarding neck dissection and parotid gland management. We have clarified these details in the revised Methods section.

In our cohort, neck dissection and parotidectomy were performed according to tumor stage and the planned extent of temporal bone resection. For T1 cases treated with sleeve resection, neither neck dissection nor parotidectomy was performed. For T1–T2 cases undergoing LTBR, superficial parotidectomy and selective neck dissection (levels I–II) were routinely performed; no lymph node metastasis or parotid involvement was identified in any early-stage case.

For T3 and T4 tumors, total parotidectomy was performed in conjunction with STBR, and neck dissection typically included levels I–III, with level IV added when preoperative imaging suggested suspicious nodes. LTBR-based procedures in advanced cases were accompanied by superficial parotidectomy.

These clarifications have been added to the revised manuscript to clearly describe the surgical management strategy across tumor stages.

2) In surgical methods and survival outcomes section, line2. T2 cases involve bone invasion, and typically cannot be treated with sleeve resection. Please verify this.

**Response:**

We thank the reviewer for this important clarification. No T2 lesions in our cohort were treated with sleeve resection. Sleeve resection was performed exclusively in T1 cases without bone invasion, whereas all T2 tumors underwent LTBR consistent with standard oncologic principles. We have clarified this point explicitly in the revised manuscript to avoid any misinterpretation.

3) In surgical methods and survival outcomes section, line4. It would be better to clearly state that all T1 and T2 cases had negative surgical margins. In addition, a description of the surgical margins for T3 and T4 cases is also necessary.

**Response:**

We agree with the reviewer and have clarified this point in the revised manuscript. All T1 and T2 cases achieved negative surgical margins on final pathological examination, and this has now been explicitly stated in the Results section. For advanced-stage disease, surgical margin status of T3 and T4 patients is presented in Table 2 and further described in both the Results and Discussion sections.

4) It is stated that extended LTBR was performed in six T4 cases; however, Table 2 lists only three such cases. Please correct this inconsistency. In addition, Table 2 includes only 19 out of the 21 T4 cases. Furthermore, in Case 13, distant metastasis is reported. Since surgery would not be considered a curative treatment in such a case, this case should be excluded.

**Response:**

We thank the reviewer for this important observation.

First, we corrected the discrepancy regarding extended LTBR: extended LTBR was performed in two T3 cases and three T4 cases, and this has been clarified in the Results section to match Table 2.

Second, although a total of 21 patients were staged as T4, Table 2 presents 19 patients because two individuals died from causes unrelated to EAC SCC (acute pneumonia at 29 months and an unidentified medical event at 41 months). Since these deaths were not attributable to tumor progression, including these cases in the anatomical risk–factor analysis would have introduced bias; therefore, they were excluded from the detailed evaluation. This rationale has now been clarified in the manuscript.

Regarding Case 13 with lung metastasis, distant metastasis was not considered an absolute contraindication to surgery in our treatment strategy. This patient was judged to have oligometastatic disease and underwent surgical resection for loco-regional disease control in conjunction with systemic treatment for the lung metastasis. Thus, surgery was undertaken with therapeutic intent rather than palliative debulking. Corresponding clarification has been added to the Discussion section.

5) In surgical methods and survival outcomes section, line6. It is stated that all cases received postoperative CRT, but this contradicts later descriptions. This requires correction.

**Response:**

We thank the reviewer for pointing out this inconsistency. The previous wording suggesting that all T3–T4 patients received postoperative CRT was inaccurate and has now been corrected.

In the revised manuscript, the statement has been updated to:
“Most patients with T3 and T4 disease received postoperative adjuvant RT or CRT according to institutional treatment principles.”

We further clarified in the Results section that a small number of patients did not complete RT or chemotherapy because they declined treatment despite medical recommendation, rather than due to physician-determined ineligibility. No patient was excluded from adjuvant therapy by clinical judgment; all omissions occurred due to patient refusal. This distinction has now been explicitly stated to avoid misinterpretation.

Corresponding corrections have been made in both the Results section and the Surgical Methods and Survival Outcomes subsection.

6) In adjuvant therapeutic patterns and results section, line4. The authors stated the involvement of multiple institutions, but I understand this study to be based on cases from a single institution. Could you clarify what is meant by this?

**Response:**

We clarify that this is a single-institution study, with all surgical treatment and data collection conducted at our center. The reference to multiple or outside hospitals reflects the fact that many patients received adjuvant radiotherapy or chemotherapy at their local hospitals after surgery. We have revised the manuscript to clarify this point and avoid misunderstanding.

7) In adjuvant therapeutic patterns and results section, line 1-2. It is written that two patients did not receive radiotherapy, and I assume this refers to two out of the 26 T3 and T4 cases.

**Response:**

Yes, this refers to 2 of the 26 patients with T3–T4 disease. These patients did not receive postoperative radiotherapy due to poor general condition, advanced age, or treatment refusal, as clarified in the Results section of the revised manuscript.

8) I consider it an extremely favorable result that there were no recurrences in T1 and T2 cases treated with surgery alone, without postoperative radiotherapy. Although it is noted that the survival rate was 100%, please also provide information regarding local or regional recurrence, as well as any distant metastases.

**Response:**

We agree with the reviewer’s comment. In our cohort, none of the patients with T1 or T2 disease, who were treated by surgery alone without postoperative radiotherapy, experienced any local or regional recurrence or distant metastasis during the entire follow-up period. This information has been added to the Results section.

9) In an univariate analysis, only the items that showed significant differences are reported. However, all items examined, including those with non-significant results, should also be presented. Regarding the univariate analysis of age, it is unclear what comparisons were made—for example, whether it was between patients aged 65 and older versus those under 65. This should be clearly specified. Furthermore, although a multivariate analysis was subsequently performed, it is not clear how the variables included in the multivariate model were selected.

**Response:**

We thank the reviewer for this important statistical clarification.

We have revised the Methods section to specify that age was treated as a continuous variable and was not dichotomized into “young” and “old” groups. Therefore, the hazard ratio reflects the incremental risk associated with increasing age rather than group-based comparisons.

We also clarified our multivariate modeling strategy. Variables demonstrating a p-value < 0.10 in univariate analysis, together with clinically relevant factors, were selected for entry into the multivariate Cox proportional hazards model.

All variables examined in the univariate analysis, including both statistically significant and non-significant factors, are now explicitly presented in Figure 2. This has also been clarified in the Results section and the figure legend.

10) The authors cite previous studies indicating that surgical margins and perineural invasion are poor prognostic factors. However, these factors are not included in the prognostic analyses of the current study. Please clarify the reason for their exclusion, and it would be desirable to include these factors in the analysis.

**Response:**

We thank the reviewer for this important comment regarding the prognostic role of surgical margins and perineural invasion.

With respect to surgical margins, margin positivity in our cohort was not analyzed as an independent prognostic variable because it showed strong collinearity with site-specific anatomical invasion, particularly carotid artery (CA) abutment, jugular bulb (JB) abutment, and Eustachian tube involvement. In most T4 cases, ‘positive margins’ on the initial en bloc specimen resulted from tumor adherence to these critical structures rather than true unresectable disease. Consequently, margin status functioned as a surrogate for the extent of anatomic invasion rather than as an independent prognostic factor. To avoid multicollinearity and maintain stability of the multivariate model, we therefore included these anatomical invasion variables instead of surgical margin status in the Cox analysis.

Importantly, no patient was left with gross residual tumor. Following initial en bloc resection, all macroscopic residual disease was removed through additional stepwise excision guided by intraoperative frozen-section confirmation until clearance of all accessible margins was achieved. Thus, initial margin positivity did not represent incomplete oncologic treatment.

Regarding perineural invasion, clinically relevant perineural invasion in this cohort was essentially reflected by cases with facial nerve involvement. Because explicit histopathologic perineural invasion occurred almost exclusively in patients with facial nerve palsy and its independent incidence was extremely low, there was insufficient statistical power to analyze perineural invasion as a separate variable. Facial nerve involvement was therefore used as its clinical surrogate in the prognostic analyses.

These clarifications have been added to the Methods and Discussion sections of the revised manuscript.

11) In cases with carotid artery invasion, achieving complete resection is generally difficult unless the carotid artery is sacrificed. However, in the present study, five such cases with carotid artery involvement are included. Since these cases are inherently challenging to treat with curative surgery, they would be considered to meet the exclusion criteria.

**Response:**

We appreciate the reviewer’s concern regarding cases with apparent carotid artery (CA) involvement and the feasibility of curative surgery.

In our cohort, all cases categorized as having CA involvement represented tumor abutment to the vascular surface rather than true intramural or intraluminal invasion of the artery. None of the patients demonstrated destructive infiltration of the carotid artery wall that would have required vascular sacrifice or indicated unresectable disease. Therefore, these cases did not meet exclusion criteria and were treated with curative intent.

In such cases, en bloc temporal bone resection was initially performed, followed by additional targeted excision and careful scraping of adherent tumor tissue from the CA surface until macroscopic clearance was achieved. No patient was left with gross residual disease after surgery. Thus, these procedures were undertaken as definitive surgical treatment rather than palliative debulking.

Although all patients with CA abutment ultimately died, this reflects the aggressive biological behavior and extensive skull-base involvement characteristic of advanced T4 disease, rather than technical futility or inappropriateness of surgical intervention.

Corresponding clarification has been added to the Discussion section of the revised manuscript.

12) In the discussion, the authors emphasize the importance of achieving negative surgical margins and en bloc resection. However, this paper does not provide any data on margin status or whether en bloc resection was performed, and merely cites previous studies. At the very least, these data should be presented, and the study should demonstrate that these factors are indeed important prognostic indicators in the current cohort.

**Response:**

We thank the reviewer for this important comment.

We have now explicitly presented both the performance of en bloc resection and detailed surgical margin status within our cohort. In the revised Results section, we clarify that all procedures were planned as en bloc resections, and complete en bloc resection was achieved in all T1–T3 cases. For T4 tumors, initial en bloc resection was followed by additional stepwise excision guided by frozen-section confirmation when critical adjacent structures were involved.

Margin status data for all T4 patients have been added to Table 2, demonstrating that negative margins were achieved on the initial en bloc specimen in 5 cases, while 14 cases showed positive initial margins related primarily to abutment of adjacent structures such as the carotid artery, jugular bulb, or Eustachian tube. Importantly, no patient was left with gross residual tumor following additional intraoperative excision.

Furthermore, our multivariate Cox analysis demonstrates that anatomical invasion—particularly carotid artery abutment—is an independent prognostic factor for survival. Surgical margin positivity was therefore analyzed indirectly through these invasion patterns as surrogate variables to avoid multicollinearity.

These additions now provide direct cohort-level evidence supporting both the importance of en bloc resection and margin clearance in outcome prediction within the current study population.
